# Peer Review of “The Performance of DeepSeek R1 and Gemini 3 in Complex Medical Scenarios: Comparative Study”

**DOI:** 10.2196/96223

**Published:** 2026-04-27

**Authors:** Ziyu Wang

**Affiliations:** 1 University of California, Irvine, Irvine, CA, United States

**Keywords:** large reasoning model, LRM, large language model, LLM, accuracy, medical scenario, DeepSeek R1, Gemini 3

*This is a peer-review report for “The Performance of DeepSeek R1 and Gemini 3 in Complex Medical Scenarios: Comparative Study.”*

## Round 1 Review

### General Comments

This is a timely and well-structured paper [1] that investigates the application of DeepSeek R1, a state-of-the-art large reasoning model, in the medical domain using the Multitask Language Understanding Pro (MMLU-Pro) benchmark. This paper presents a follow-up evaluation of the DeepSeek R1 large reasoning model on open-ended medical scenarios from the MMLU-Pro benchmark. The study finds that DeepSeek R1 achieves a high accuracy of 92% without multiple-choice options, demonstrating its potential utility in more realistic clinical settings. The paper is timely and relevant, with strong empirical results and clear motivation. However, it would benefit from revisions to improve clarity, contextual grounding in existing work, and methodological detail. The authors may also consider citing recent work that examines the questioning strategies of large language models (LLMs) in clinical dialogues to better position this study in the broader landscape.

The study is commendable for its effort in combining expert validation with benchmark testing and highlighting both performance and interpretability aspects. The paper is generally well-written, informative, and relevant to the research community on artificial intelligence (AI) in health care.

However, before being suitable for publication, several important revisions are required. These include expanding the related work section to better situate the contribution of current research efforts, addressing some methodological limitations more transparently, and improving the robustness and generalizability of conclusions. Thus, I recommend revision and re-review.

### Specific Comments

#### Major Comments

1. While the paper references MMLU, MedQA, and some domain-specific LLM evaluations, it lacks a deeper discussion on recent approaches to questioning capabilities and long-context understanding in medical AI. Two notable papers should be included. First, “HealthQ: Unveiling Questioning Capabilities of LLM Chains in Healthcare Conversations” by Wang et al [2]. This paper presents a benchmarking framework focusing on the inquiry and elicitation capacity of LLM chains, which directly relates to the “reasoning” and prompt design aspects discussed here. Second, ”Context Clues: Evaluating Long Context Models for Clinical Prediction Tasks on EHR Data” by Wornow et al [3]. This study highlights how context windows and task framing affect LLM performance on clinical reasoning—relevant for understanding how question complexity and format might interact with LLM accuracy.

The paper could be strengthened by referencing more recent work on prompting and questioning strategies in clinical LLM applications. The paper would benefit from referencing Wang et al [2], which evaluates LLM chains’ ability to optimize questions through reflection and prompting. This is relevant to the current paper’s interest in open-ended diagnostic reasoning and LLM behavior in clinical settings.

2. Although the DeepSeek R1 model is rigorously evaluated against MMLU-Pro, there’s a lack of direct performance comparison to other LLMs (eg, MedPaLM, GPT-4, Claude) on the same dataset or medical scenarios. Even informal or partial benchmarks would help contextualize the model’s effectiveness. Also, the novelty should be better emphasized—is this the first comprehensive large reasoning model evaluation on MMLU-Pro’s health subset?

3. The paper rightly points out issues with cueing and “testwiseness” in multiple-choice questions but doesn’t propose concrete mitigations. The planned future work of testing without answer choices is excellent—consider incorporating a small pilot of this now or discussing expected outcomes in more depth. Also, the limitations of using only 162 scenarios across many specialties could be made more transparent, especially regarding statistical robustness and specialty-specific insights.

4. The study uses a fixed prompt but does not explore or discuss the impact of prompt variations, which may influence results in open-ended tasks.

5. While the discussion of biases and failure modes is helpful, a more structured breakdown of error types and their frequency would improve the interpretability of findings.

6. The discussion on reasoning steps and transparency is insightful but could be expanded to address recent concerns about the faithfulness of chain-of-thought outputs.

#### Minor Comments

7. Model latency and usability: while the latency of DeepSeek is acknowledged, it’s not contextualized with respect to potential clinical utility or workflow integration. A brief paragraph on practical deployment implications would strengthen the discussion.

8. Citation formatting: ensure all references (especially web-based ones like Perplexity and PromptHub) are consistently formatted and maintained in the reference list.

9. Future directions could be made more actionable by suggesting benchmark expansions with real patient data or multimodal inputs.

## Round 2 Review

My comments have been addressed.

## Abbreviations

AI: artificial intelligence
LLM: large language model
MMLU-Pro: Multitask Language Understanding Pro
